# Bacterial isolation from internal organs of rats (*Rattus rattus*) captured in Baghdad city of Iraq

**DOI:** 10.14202/vetworld.2019.119-125

**Published:** 2019-01-22

**Authors:** Nagham Mohammed Ayyal, Zainab Abdulzahra Abbas, Abdulkarim Jafar Karim, Zainab Majid Abbas, Karima Akool Al-Salihi, Jenan Mahmood Khalaf, Dunya Dhafir Mahmood, Eman Abdullah Mohammed, Rawaa Saladdin Jumaa, Dhuha Ismaeel Abdul-Majeed

**Affiliations:** 1 Unit of Zoonotic Diseases, College of Veterinary Medicine, University of Baghdad, Baghdad, Iraq; 2 Department of Pathological Analysis, Babylon Technical Institute, Al-Furat Al-Awsat Technical University, Babylon, Iraq; 3 Department of Internal and Preventive Medicine, College of Veterinary Medicine, Al-Muthanna University, Al-Muthanna, Iraq; 4 Department of Internal and Preventive Medicine, College of Veterinary Medicine, University of Baghdad, Baghdad, Iraq; 5 Department of Parasitology, College of Veterinary Medicine, University of Baghdad, Baghdad, Iraq; 6 Department of Microbiology, College of Veterinary Medicine, University of Baghdad, Baghdad, Iraq

**Keywords:** bacteria, different organs, *Escherichia coli* O157:H7, *Pseudomonas aeruginosa*, rat, urine

## Abstract

**Aim:**

Rats are accused in disseminating many zoonotic diseases. This study aimed to isolate and identify bacteria from internal organs of rats captured in Baghdad City, Iraq.

**Materials and Methods:**

A total of 120 black rats (*R. rattus*) were trapped from different areas in Baghdad city. Rats were kept in individual plastic cages for 3 h before euthanizing. Deep pharyngeal swab, intestinal content, urine, and pieces of the liver and spleen, lung, kidney, and brain were obtained aseptically. The specimens were inoculated into peptone water and incubated at 37°C for 24 h for enrichment. A loopful of each specimen was then subcultured onto MacConkey Agar, Blood Agar, and Mannitol Salt Agar. CHROMagar O157 H7 and CHROMagar Listeria were used to detect *Escherichia coli* 157:7 and *Listeria* spp., respectively. Biochemical tests on analytical profile index, microscopic examination, and commercial kit for latex agglutination test for serotyping *E. coli* O157:H7 were used.

**Results:**

Mixed bacterial isolates were recorded as 116, 52, 36, 28, 18, 6, and 4 from intestinal contents, deep pharyngeal, liver and spleen, urine, lung, brain, and kidney, respectively. Microorganisms included *E. coli*, *Staphylococcus aureus*, *Streptococcus* spp., *Bacillus* spp., *Pseudomonas aeruginosa*, *Citrobacter freundii*, *Proteus vulgaris*, *E. coli* *O157:H7*, *Enterobacter cloacae*, *Listeria* spp., *Klebsiella* spp., *Ochrobactrum anthropi*, *Aeromonas* spp., *Brucella* spp., *Pseudomonas fluorescens*, *Escherichia fergusonii*, *Micrococcus* spp., *Morganella* spp., *Proteus mirabilis*, *Pseudomonas luteola*, and *Streptobacillus* spp. The highest bacterial prevalence (88; 73.33%) was recorded for *E. coli*, where 68 isolates were identified from the intestinal contents. Of these, four isolates were *E. coli* O157:H7.

**Conclusion:**

Rats are important carriers and transmitters of a number of pathogens and can disseminate these microorganisms to humans and animals.

## Introduction

Rats, not like all other mammals, inhabit all continents wherever human is present. Their serious role in dispersing diseases arises from contamination of food and living places. They are incriminated in transmitting several zoonotic and non-zoonotic microorganisms causing diseases in humans and animal due to their tendency to invade houses with subsequent contamination of foods [1]. These microorganisms include many species of bacteria, fungi, viruses, rickettsia, protozoa, helminths, and finally, their ectoparasites such as fleas [2-5]. Many of these had rarely or never previously been investigated, for example, *Cryptosporidium*, *Pasteurella*, *Listeria*, *Yersinia*, and *Coxiella* [6,7]. Most of these organisms are not directly responsible for epidemics, while others such as *Yersinia pestis*, *Streptococcus moniliformis*, and *Salmonella typhimurium* are incriminated in outbreaks of plaque, Haverhill fever, and salmonellosis, respectively [8,9]. Rats are capable of carrying and shedding *Escherichia coli* [10-12], and it has been isolated from several wildlife species including rodents, bats, and farmed and wildlife animals [13,14].

Closer contact with rats means more diseases. Rats transmit diseases directly or indirectly. They are incriminated for deaths more than any other causes. Threat to human health is well recognized when potentially life-threatening diseases that currently have no specific treatment, cure, or vaccine [15], for example, Hantavirus pulmonary syndrome, leptospirosis, cutaneous leishmaniasis, toxoplasmosis, rat-bite fever, plague, salmonellosis, tularemia, lymphocytic choriomeningitis, plague, and Colorado tick fever are harbored by rats [6,16].

This study aimed to isolate and identify bacteria from internal organs of rats captured in Baghdad City, Iraq.

## Materials and Methods

### Ethical approval

The experiment was carried out in the Laboratory of Zoonotic Diseases, College of Veterinary Medicine, University of Baghdad, from September 1 to September 30, 2015, and approved by the Animal Care and Use Committee (Approval No. 1593/28 August 2015).

### Animals

A total of 120 black rats (*Rattus rattus*) were trapped from different areas in Baghdad city. Rats were kept in individual plastic cages for 3 h before euthanized. Pellets and water were supplied *ad libitum*. The rats were administered 0.1 mL of 9:1, Ketamine + Xylazine per 100 g/BW intramuscularly [17].

### Collection and processing of samples

Deep pharyngeal swab, intestinal content, urine, and pieces of the liver and spleen, lung, kidney, and brain were obtained aseptically. The specimens were inoculated into peptone water and incubated at 37°C for 24 h for enrichment. A loopful of each specimen was then subcultured onto MacConkey Agar, Blood Agar, and Mannitol Salt Agar. CHROMagar O157 H7 and CHROMagar Listeria were used to detect *E. coli* 157:7 and *Listeria* spp., respectively [18,19].

### Identification of bacteria

Identification of bacteria was confirmed using biochemical tests on analytical profile index (Analytab Products, BioMerieux Canada, St. Laurent, Quebec) strips and microscopic examination. A commercial kit (Wellcolex *E. coli* O157:H7, Remel) for latex agglutination test was used to detect both the somatic antigen O157 and the flagellar antigen H7 for serotyping *E. coli* O157:H7. This test was performed according to the manufacturer’s instructions.

## Results

Mixed bacterial isolates recorded 116, 52, 36, 28, 18, 6, and 4 were identified from intestinal contents, deep pharyngeal, liver and spleen, urine, lung, brain, and kidney, respectively (Table-1). The highest bacterial prevalence (88; 73.33%) was recorded for *E. coli*, where 68 isolates were identified from the intestinal contents. Of these, four isolates were *E. coli* O157:H7. Notably, *Staphylococcus* spp. (50; 41.66%), *Streptococcus* spp. (18; 15%), *Bacillus* spp. (14; 11.66%), and *Pseudomonas aeruginosa* (12; 10%) together comprised a high prevalence rate. *Proteus* spp., *Listeria* spp., *Klebsiella* spp., *Brucella* spp., and many other bacteria were isolated in low frequency, as shown in Table-1.

**Table-1:** Bacterial isolates from the internal organs of captured rats.

Isolated bacteria	Intestinal content	Deep pharyngeal swab	Liver and spleen	Urine	Lung	Brain	Kidney	Total prevalence (%)
*Escherichia coli*	68	0	10	10	0	0	0	88 (73.33)
*Staphylococcus aureus*	6	30	0	6	8	0	0	50 (41.66)
*Streptococcus* spp.	2	6	0	2	4	4	0	18 (15.00)
*Bacillus* spp.	6	0	0	4	4	0	0	14 (11.66)
*Pseudomonas aeruginosa*	0	4	8	0	0	0	0	12 (10.00)
*Citrobacter freundii*	4	0	4	2	0	0	0	10 (8.33)
*Proteus vulgaris*	0	4	2	4	0	0	0	10 (8.33)
*E. coli O157:H7*	8	0	0	0	0	0	0	8 (6.66)
*Enterobacter cloacae*	4	2	0	0	0	0	2	8 (6.66)
*Listeria* spp.	6	0	0	0	0	0	0	6 (5.00)
*Klebsiella* spp.	6	0	0	0	0	0	0	6 (5.00)
*Ochrobactrum anthropi*	0	0	4	0	0	2	0	6 (5.00)
*Aeromonas* spp.	0	0	4	0	0	0	0	4 (3.33)
*Brucella* spp.	0	0	4	0	0	0	0	4 (3.33)
*Pseudomonas fluorescens*	0	2	0	0	0	0	2	4 (3.33)
*Escherichia fergusonii*	2	0	0	0	0	0	0	2 (1.66)
*Micrococcus* sp.	0	0	0	0	2	0	0	2 (1.66)
*Morganella* spp.	2	0	0	0	0	0	0	2 (1.66)
*Proteus mirabilis*	2	0	0	0	0	0	0	2 (1.66)
*Pseudomonas luteola*	0	2	0	0	0	0	0	2 (1.66)
*Streptobacillus*	0	2	0	0	0	0	0	2 (1.66)
Total	116	52	36	28	18	6	4	

## Discussion

Rats have been incriminated as the carriers of many pathogenic bacteria [20]. Results of this study indicated that rats harbor bacterial organisms, which are potentially pathogenic to humans or animals. Our findings recorded the isolation of 20 different genera of bacteria from different organs of the rat including *E. coli*, *Listeria*, *Brucella*, *P. aeruginosa*, *Staphylococcus aureus*, *Streptococcus* spp., and *Streptobacillus* (Table-1). *E. coli* is a normal microflora of the gut and causes gastrointestinal disruption. The more pathogenic serotype, *E. coli* O157:H7, has emerged as a major foodborne zoonotic pathogen responsible for the hemorrhagic colitis and hemolytic uremic syndrome in human [21]. In this study, the prevalence of *E. coli* and *E. coli* O157:H7 was 73.33 and 6.66%, respectively. Moine *et al*. [22] isolated these organisms from wild rodents. Previous recorded prevalence in intestinal content reached 47.56% [21], 61.8% [14], and 83.8% [12]. These data supported our findings. On the other hand, *Escherichia fergusonii*, an emerging zoonotic potential pathogen, was isolated (1.66%) from intestinal content (Table-1). It is incriminated in cystitis [23], cholangiosepsis [24], wound infections, bacteremia, diarrhea, and pleural infections in human [24-26], enteritis in broiler chickens [27,28] and ostriches [29], and diarrhea in farm animals [30-32]. Pure *E. fergusonii* growth was verified from postmortem intestinal sample, lung, liver, and kidney [30].

*Staphylococci* are diverse ubiquitous opportunistic colonizers of human epithelia involved in nosocomial infections that cause diseases of major importance in both human and animals, ranging from minor skin infections to life-threatening bacteremia as well as septicemia [33]. Kato *et al*. [34] recorded 18.1% for the prevalence of *S. aureus* from rats. Compared it to our findings (41.66%), this high prevalence may be due to the different sites of isolation included in our study. This agreed with Al-Edany [35] who recovered *S. aureus* from the respiratory tract of rats and the results of Khalaf *et al*. [36] who isolated *Staphylococcus* spp. and methicillin-resistant *S. aureus* (MRSA) from deep pharyngeal, feces, and urine. Globally, MRSA persists to be a big threatening concern in the emergency department patients [37-39].

*Streptobacillus* was isolated from deep pharyngeal swab (1.66%) of rats in our study. This organism is part of the normal flora of the rat oropharynx, and present in rat populations worldwide. It can be transmitted to people through the bite of an infected rat causing rat bite fever, and through ingestion of food contaminated by rats, causing Haverhill fever. Infection with *S. moniliformis* can progress to septicemia, and the mortality rate may record 7-13% [26,39,40].

*P. aeruginosa* is the most important antibiotic-resistant bacteria associated with nosocomial infections causing notable morbidity and mortality [41,42] with its serious mode of transmission through tap water [43]. It is incriminated in pneumonia, septicemia, surgical wound infections, and urinary tract infections [42,44,45]. The prevalence in a study performed by Gakuya *et al*. [20] was 0.6%. Our finding reported much higher prevalence peaked to 10%. Attention should be focused on this serious increment due to the involvement of this pathogen in persistent colonization of the respiratory tract and resistance for treatment [46,47]. Unlike *P. aeruginosa*, *Pseudomonas fluorescens* is an infrequent and low virulence cause of human infections which occur mostly in contaminated blood transfusion, catheter, and peritoneal dialysis in immunocompromised patients [48]. The isolation of *P. fluorescens* from deep pharyngeal swab obtained by our results (Table-1), in addition to kidney, might be a source for contamination of water sources often leading to nosocomial outbreaks [49]. Sporadic clinical infection of *Pseudomonas luteola* in which septicemia, meningitis, endocarditis, or peritonitis observed following peritoneal dialysis [50-53] or pneumonia and bacteremia occurred sequenced to multiple tick bites and leg ulcers [54]. Although our finding was confined with only two cases of *P. luteola* from deep pharyngeal swab, this might be an important source for contaminating food and utensils.

*Listeria* spp., in particular, *L. monocytogenes*, is the most important species involved in a global human health hazard affecting mammals, poultry, fish, and ticks [55,56]. The previous study reported high incidence reached 39.9% of *L. monocytogenes* in meat and its related utensils with a mortality rate peaked to 24% among human [57]. This organism was also reported in wild animals [58]. Its prevalence in the intestinal content of rats varied ranging from 6.5 to 77.8% [59,60] wherein our study was 5%. The frequent isolation of Listeria from rats suggests the possibility of rats as a reservoir of *Listeria* spp. and the continual environmental contamination. Another Gram-negative bacteria, *Brucella abortus*, was isolated from rats with active brucellosis trapped from a cattle farm and suggests that cattle are an important source of infection for rats [61]. Real incidence of brucellosis is variable due to underreporting and difficult diagnosis, and it can be up to 5 times higher. *B. abortus* as well as *Brucella melitensis* has been isolated from rats [62,63]. Rats play a crucial role in vertical transmission of brucellosis and may become potentially latent carriers providing a reservoir for future transmission [63]. Our findings revealed a prevalence of *Brucella* at 3.33% affecting liver and spleen, although species have not been identified.

Streptococci may, or not, be associated with diseases in rats [64]. Unlikely, in humans, *S. pyogenes* colonizes the oropharynx [65] causing many diseases with wide range of symptoms manifested by acute rheumatic fever, carditis, and valvulitis [66,67]. Moreover, streptococci are commensal opportunistic pathogens of the human vaginal, intestinal, and respiratory tracts causing sepsis, pneumonia, or meningitis [66-68]. Although species identification for *Streptococcus* is not carried out in our study, its prevalence (15%) in intestinal contents, deep pharynx, urine, and lung tissues predicted the transmission of diseases through rat droppings or interfering with human food. Many previous studies referred to the isolation of *Streptococcus* mutans from the oral cavity of human and rats [69,70].

Our findings showed a cumulative prevalence (10%) for *Proteus vulgaris* and *Proteus mirabilis*. Although the trend was recorded for the former, the disruptive capability attributed to the latter. *Proteus mirabilis*, occasionally, can be a primary pathogen, concomitantly with another pathogen, or alone can cause ascending pyelonephritis [71]. It is mainly in soil and the gastrointestinal tract, giving rise to opportunistic disease in children and the elderly [72]. It persists in the rat kidney for up to 8 weeks and induces many morphological changes including tubular atrophy and interstitial fibrosis resulting in chronic pyelonephritis and may extend to the prostates [72,73]. It can cause serious renal damage, such as acute pyelonephritis, renal stone, and bacteremia [73]. Similar to the prevalence of *P. vulgaris*, *Citrobacter freundii* record (8.33%) is considered a serious alert for its destructive role.

*Citrobacter* spp. are well known to be unique in their frequent association with brain abscess formation and neonatal meningitis [72]. After invading and trancytosing them, *Citrobacter freundii* is able to replicate within human brain microvascular endothelial cells causing meningitis and brain abscess and resulted in unacceptable rates of morbidity and mortality in neonates [74].

Many opportunistic pathogens, for example, *Klebsiella*, *Enterobacter*, and *Pseudomonas* have become increasingly relevant as the causative agents of clinical diseases and pathological lesions in laboratory animals [72,75]. As *K. pneumoniae* can cause severe fatal pyogenic pneumonia in humans, with a lesser extent in *K. oxytoca*, it serves experimentally as a model for many diseases, while *K. oxytoca* is infrequent naturally occur [75-77]. Our finding regarding *Klebsiella*, despite its species, reported six cases from intestinal content, putting the risk of contaminating the surroundings by rat droppings is highly predicted. Klebsiella infections in rats are widely documented experimentally as well as naturally [72,78-81], and antibiotic resistance is very common in human as well as rats [7-77,82]. Another Gram-negative bacteria, *Enterobacter cloacae*, a normal gut flora, is responsible for increasing nosocomial infections and antibiotic resistance. This microorganism causes sepsis and meningitis mainly in immunocompromised patients [83-85]. Our records regarding *E. cloacae* reached 6.66% from intestinal content, and deep pharyngeal swab and kidney predispose the contamination of food and places. The threats of genus *Enterobacter* may extend to high mortality rates in premature infants [85]. Hunter *et al*. [86] used rat model for an experimental *Enterobacter* infection.

Other opportunistic bacteria such as *Morganella* sp., *Aeromonas* sp., *Ochrobactrum anthropi*, and *Micrococcus sp*. are infrequently causing disease in healthy individuals and usually occur in the environment as normal flora [87-89]. The risk of these bacteria is in their resistance to antibiotics [90,91], residing as emerging pathogens of low virulence. Although some strains of *M. morganii* are enteropathogenic [92], most bacteremic cases (0.69% up to 3.6% among nosocomial infections) were opportunistic community-acquired infections [93,94]. Mostly, clinical infections involve the urinary tract, skin and soft tissue, hepatobiliary infection, peritonitis, septic arthritis, pericarditis, meningitis, otitis media, gastroenteritis, tubo-ovarian abscess, and neonatal sepsis [93,95]. Rats under the influence of antibiotics are able to shed *Proteus mirabilis* and Morganella morganii in feces [96]. They, in concomitant with *Aeromonas* sp., are usually involved in summer diarrhea in healthy individuals [92,97-99]. Regarding *Aeromonas* sp., a common zoonotic pathogen mainly of aquatic animals [100], it produces enterotoxin and alpha- and beta-hemolysin, resulting in hemorrhagic enteritis, and usually develops antibiotic resistance [91]. Our records 5.00, 3.33, 1.66 and 1.66% for *O. anthropi*, *Aeromonas* sp., *Micrococcus* sp. and *Morganella* sp., respectively, are in accordance with many authors who stated the role of rat in contaminating the environment and dispersing diseases [20,96,101,102]. Nosocomial infections through dialysis fluids and contaminated hospital water supplies, mostly by carrier rats, suggest a good indicator for bacterial pollution of fresh water and promote spread of nosocomial infection [87-89,102].

In this study, we reported mixed bacterial isolates from urine, deep pharyngeal swab, and many organs of rats which are usually incriminated in zoonotic diseases. Some of these bacteria, for example, *O. anthropi* have not been reported previously from captured rats.

## Conclusion

Findings of our microbiological investigation concluded that rats are important carriers and transmitters of a number of pathogens and can disseminate these microorganisms to humans as well as animals.

## Authors’ Contributions

AJK, JMK, ZAA, NMA, and ZMA designed the experiment. AJK, JMK, and NMA interpreted the data. AJK and NMA wrote the draft of manuscript. ZMA, DDM, KAA, and EAM captured rats. RSJ, DIA, and AJK euthanized the rats. All researchers participated in dissecting and sample collections. NMA, ZAA, AJK, JMK, and ZMA were responsible for culturing and biochemicals. AJK edited the manuscript.
